# A Chinese adaptation of the Patient Health Questionnaire for Adolescents (PHQ-A): factor structure and psychometric properties

**DOI:** 10.1186/s12888-024-05783-3

**Published:** 2024-04-30

**Authors:** Yu-Qi Chen, Xiao-Jie Huang, Fan Yang, Jing-Jing Yang, Jing Zhong, Kai-Min Yao, Jing-Xiao Kuang, Ming-Zhi Xu

**Affiliations:** 1 School of Public Health, Southern Medical University, Guangzhou, Guangdong People’s Republic of China; 2 Guangdong Mental Health Center, Guangdong Provincial People’s Hospital (Guangdong Academy of Medical Sciences), Southern Medical University, Guangzhou, Guangdong 510120 People’s Republic of China

**Keywords:** Depression, PHQ-A, Adolescents, Psychometric properties, Factor structure, Cut-off value

## Abstract

**Background:**

To examine the factor structure and psychometric properties of the Patient Health Questionnaire for Adolescents (PHQ-A) in Chinese children and adolescents with major depressive disorder (MDD).

**Methods:**

A total of 248 MDD patients aged between 12 and 18 years were recruited and evaluated by the Patient Health Questionnaire for Adolescents (PHQ-A), the Center for Epidemiological Survey Depression Scale (CES-D), the Mood and Feelings Questionnaire (MFQ), and the improved Clinical Global Impression Scale, Severity item (iCGI-S). Thirty-one patients were selected randomly to complete the PHQ-A again one week later. Confirmatory factor analysis (CFA) was used to test the construct validity of the scale. Reliability was evaluated by Macdonald Omega coefficient. Pearson correlation coefficient was used to assess the item-total correlation and the correlation of PHQ-A with CES-D and MFQ respectively. Spearman correlation coefficient was used to assess test-retest reliability. The optimal cut-off value, sensitivity, and specificity of the PHQ-A were achieved by estimating the Receiver Operating Characteristics (ROC) curve.

**Results:**

CFA reported adequate loadings for all items, except for item 3. Macdonald Omega coefficient of the PHQ-A was 0.87. The Spearman correlation coefficient of the test-retest reliability was 0.70. The Pearson correlation coefficients of the PHQ-A with CES-D and MFQ were 0.87 and 0.85, respectively (*p* < 0.01). By taking the iCGI-S as the remission criteria for MDD, the optimal cut-off value, sensitivity and specificity of the PHQ-A were 7, 98.7%, 94.7% respectively.

**Conclusion:**

The PHQ-A presented as a unidimensional construct and demonstrated satisfactory reliability and validity among the Chinese children and adolescents with MDD. A cut-off value of 7 was suggested for remission.

## Introduction

Major Depression Disorder (MDD) is a prevalent mental disorder. Studies in various countries have reported its lifetime prevalence of, for example, 20.5% in Chile [1], 21% in France [2], 6.7% in South Korea [3] and 3.4% in China [4]. In 2008, the World Health Organization (WHO) ranked MDD as the third leading contributor to the global burden of disease, predicting that it would ascend to the primary position by 2030 [5, 6]. Among Chinese children and adolescents, depressive symptoms have become more prevalent and the pooled prevalence of depressive symptoms among them was 22.2% [7]. Depression often leads to a cascade of consequences, including suicidal ideation, school dropout, behavioral disturbances, and substance abuse among children and adolescents [8]. Additionally, 10%-25% of patients with mild symptoms are at risk of scoring in the severe range over one to three years without intervention in time [9]. Adolescence is frequently regarded as a pivotal period for the early identification and prevention of adult depression [10], highlighting the vital importance of prompting diagnosis and intervention to prevent the onset of depression in adolescents.

Using simple and efficacious screening tools is an effective way to improve the detection of mental disorders [11]. The US Preventive Services Task Force (USPSTF) recommended to adopt screening tools like the Patient Health Questionnaire for Adolescents (PHQ-A) and the primary care version of the Beck Depression Inventory (BDI) for the identification of depression among adolescents in primary care settings [12]. Self-rating scales are simple and efficacious screening tools to screen depression among adolescents. It can not only help clinicians to quantify the patients' subjective feelings, but also enable patients understand the severity of their distress. Meanwhile, the Measurement-based care (MBC), which focus on the periodical assessments of the treatment in the process of quality controls, has been widely recommended in psychiatric practice. The MBC allows clinicians to make personalize treatment decisions for the patients, thereby improving the adoption of appropriate treatment strategies, reducing treatment resistance, and increasing treatment quality [13].

In clinical practice, the depression assessment scales used for children and adolescents mainly include the Children's Depression Inventory (CDI), the Center for Epidemiological Studies Depression Scale (CES-D), the primary care version of the Beck Depression Inventory (BDI), the Mood and Feelings Questionnaire (MFQ) and the Patient Health Questionnaire for Adolescents (PHQ-A/PHQ-9 M) [12, 14].

The PHQ-A/PHQ-9 M is adapted from the 9-item Patient Health Questionnaire-9 (PHQ-9). The PHQ-9 was initially developed by Kroenke and Spitzer in 2001 for assessing depression in adult primary care and then was extended to adolescent depression [15–18]. The advantage of the PHQ-9 was that it exclusively focuses on the 9 diagnostic criteria of MDD in the fourth edition of the Diagnostic and Statistical Manual of Mental Disorders (DSM-IV), which makes it more specific for major depression and may accurately discriminate depression from anxiety or even general psychological distress [19]. However, previous studies reported that the specificity of the PHQ-9 was lower when used in adolescents, which could lead to an increased likelihood of false positive rate [16, 20], suggesting that the PHQ-9 may not be the most suitable screening tool for assessing depression in adolescents. This could potentially be attributed to the specific features of adolescent depression that may differ from adult depression. Although some of the symptoms of MDD may be similar for adults and adolescents, its clinical feature and prominence may be different [21]. Irritability is an impairing clinic manifestation that refers to easy annoyance and touchiness, which has been the most frequently reported symptom in adolescent depression [21, 22]. Although the DSM-IV identifies irritability as a characteristic of adolescent depression, it states that ‘it is not a criterion for major depressive episode’ [21]. Yet both the DSM-5 and the 11th edition of the International Classification of Diseases (ICD-11) include the criteria to specifically define MDD for adolescent with the statement ‘Depressed mood (subjective or observed) can be irritable mood in children and adolescents’ [23], suggesting the importance of irritability on the diagnosis of MDD among adolescents. Comparing to the PHQ-9, the PHQ-A adds ‘irritability’ in the description of item 1, which is the major revision. Moreover, two minor revisions include that the order of item4 (Fatigability) and item5 (Appetitive problems) reverse, and the PHQ-A added ‘school work’ in item7 (Concentration problems), which adapts to children`s daily activities. With these revisions, the PHQ-A may be more appropriate for screening depression in adolescents and adjusts the DSM-5 and ICD-11 diagnosis better.

The English version of the PHQ-A showed satisfactory sensitivity, specificity and overall diagnostic accuracy and its reliability and validity was proved satisfactory [24, 25]. The PHQ-A has been translated into other languages such as Urdu, Thai, Portuguese and Arabic [26–29]. All of these translations demonstrated satisfactory reliability and validity in relative countries and populations. In the Urdu, Thai, and Arabic version, only one factor was extracted by exploratory factor analysis (EFA). Furthermore, the unidimensional factor structure is verified by confirmatory factor analysis (CFA) in the Urdu and Arabic version [26–29]. Therefore, the PHQ-A is a promising screening tool and merits further evaluation among adolescents in China.

In this study, we hypothesized that the PHQ-A would fit into a unidimensional structure and demonstrate good psychometric properties among Chinese children and adolescents with MDD. We would test the hypotheses and recommend a cut-off value for remission.

## Method

### Participants

Participants were recruited from the outpatient and inpatient departments of Guangdong mental health center, Guangdong Provincial People's Hospital from December 2022 to June 2023. This study was authorized by the Clinical Research Ethics Committee of Guangdong Provincial People’s Hospital (No. KY-Z-2022-062-01), and carried out based on the Declaration of Helsinki. Informed consent was obtained in all patients. The enrollment criteria were as follows: (1) sign an informed consent form to participate in the research; (2) be between 12 and 18 years old; (3) meet the diagnostic criteria of MDD in the DSM-5; (4) be able to understand the questionnaire and report their status. The exclusion criteria were: (1) diagnosed with bipolar disorder or other severe mental disorders such as schizophrenia; (2) a history of alcoholism, drug abuse, or serious physical diseases; (3) diagnosed with mental retardation. Recommendations in the literature suggested a minimum sample size for CFA of around 100-250 observations [30].

Patients who met the conditions for enrollment entered the study group. Their demographic data (age, birth date, sex, ethnicity, education year, living conditions, insurance type, age of first onset, and family history of mental illness) were collected. Then they completed the scales in a quiet room. 31 patients of the participants were randomly selected and retested 7 days after the first test to assess the test-retest reliability of the PHQ-A.

### Instruments

#### The Patient Health Questionnaire for Adolescents (PHQ-A)

The PHQ-A is adapted from the PHQ-9 for measuring depression among adolescents. It is a 9-item self-report scale developed by Spitzer and Johnson in 2002. The items are rated on a 4-point Likert scale: 0 (none), 1 (several days), 2 (more than half the days) and 3 (nearly every day). The total score ranges from 0 to 27. Higher scores indicate greater symptom severity. The PHQ-A is an acceptable and efficient screening tool in primary care settings [24].

In this study we translated the PHQ-A and the translating process were as follows. Firstly, one psychiatrist and two psychologists who were good at English translated the original version of PHQ-A from English to Chinese. Secondly, after translating it back into English, they made adjustment until it was almost the same as the original one. Thirdly, we choose 10 individuals at random to participate in a pilot test. Based on the feedback from the test, the final Chinese translation was confirmed.

#### The Center for Epidemiological Survey Depression Scale (CES-D)

The CES-D was used as the criterion scale in our study. It is a 20-item self-rating scale developed by Radloff in 1977 [31]. The CES-D are rated on a 4-point Likert scale. The item 4, the item 8, the item 12 and the item 16 are reversely scored. The total score ranges from 0 to 60, with higher scores suggesting greater symptom severity. The Chinese version of CES-D is a reliable and valid screening tool for Chinese adolescents [32–34].

#### The Mood and Feelings Questionnaire (MFQ)

The Mood and Feelings Questionnaire (MFQ) was used as another criterion scale in our study. The child self-report MFQ developed by Angold in 1995 is a 33-item scale [35]. It was developed to measure depression among children and adolescents. The child self-report MFQ is rated on a 3-point Likert scale: 0 (not true) to 2 (true), with higher scores suggesting greater depressive symptom severity. The child self-report MFQ is a reliable and valid screening tool for Chinese children and adolescents [36].

#### The improved Clinical Global Impression Scale, Severity item (iCGI-S)

The Clinical Global Impression (CGI) scale is a classic scale to rate the clinician’s overall impression upon a patient’s current condition [37]. The iCGI-S is an item of improved CGI that rates the severity of depression symptoms. It is rated by clinicians on a 7-point Likert scale: 1 = normal, 2 = borderline ill, 3 = mildly ill, 4 = moderately ill, 5 = markedly ill, 6 = severely ill, and 7 = among the most extremely ill. iCGI-S = 1 was commonly used to differentiate patients in remission from those are not and acted on establishing the cut-off score [38].

### Statistical analysis

EpiData3.1 was used for data entry, AMOS 24.0 for verifying the factor structure of the PHQ-A and SPSS 25.0 for data processing and analysis. Confirmatory factor analysis (CFA) was used to verify the unidimensional structure of the PHQ-A. Tucker-Lewis Index (TLI), Comparative Fit Index (CFI), Goodness of Fit Index (GFI) and Root Mean Square Error of Approximation (RMSEA) were selected as indicators. Based on the existing recommendations, acceptable model fit was considered if the CFI, GFI and TLI were greater than 0.9 and the RMSEA was smaller than 0.10 [39–42]. Macdonald Omega coefficient was used to evaluate the internal consistency reliability. Spearman correlation coefficient was applied to calculate the test-retest reliability. Item-total correlation and criteria validity were estimated via Pearson correlation coefficient. Due to the sample size, Kruskal–Wallis H Test was used to compare the total scores of the PHQ-A with different severity of depressive symptoms classified by the iCGI-S. Receiver Operating Characteristic (ROC) curve was established on the basis of iCGI-S = 1. Area Under the Curve (AUC) and Youden’s index were calculated to evaluate the sensitivity, specificity and optimal cut-off score of the PHQ-A.

## Results

### Demographic characteristics and distribution of the PHQ-A scores

A total of 253 patients were recruited. Five patients who did not perform seriously (such as responding in a certain order of categories like ‘1,2,3,4,1,2,3,4……’ or responding rapidly and finishing the scales in an unreasonable time) were considered to be invalid responses and were eliminated. Finally, 248 patients were included with a response rate of 98.02%. As shown in Table 1, the age of the patients ranged from 12 to 18 years, with a mean age of 15.42 years (SD = 1.70). 187 patients (75.4%) were female and the other 61 (24.6%) were male. The age on their first episode ranged from 8 to 18 years, with an average of 14.60 (SD = 1.82). 212 patients (85.5%) experienced their first MDD episode. Their education years ranged from 5 to 12 years, with an average of 9.35 (SD = 1.75). The mean score of PHQ-A in females and males was 16.98 (SD = 6.16) and 16.28 (SD = 6.43) respectively, which did not differ significantly (t = 0.77 and *P* = 0.44).

**Table 1:** Demographic characteristics of the participants

	Total Sample (*n* = 248)	%
Age		
12–15	125	50.4
16–18	123	49.6
Gender		
Female	187	75.4
Male	61	24.6
First episode		
Yes	212	85.5
No	36	14.5
Ethnicity		
Han	242	97.6
Others	6	2.4
Living condition		
Alone	4	1.6
With family	231	93.2
With others	13	5.2
Insurance type		
Yes	76	30.6
No	172	69.4
Family history of mental disorders		
Yes	36	14.5
No	212	85.5
Edu year		
5–9	131	52.8
10–12	117	47.2
ICGI-S score		
1	19	7.7
≥ 2	229	92.3
Mean score	16.81	SD = 6.23

Note: *Mean score* the mean score of the PHQ-A, *SD* Standard Deviation

The mean score of the PHQ-A of the total sample was 16.81 (SD = 6.23). The score of the patients ranged from 0 to 27 with a mode of 20. According to the data, only 8 participants (3.2%) received a minimum score, while 42 (9.1%) had the maximum score of 27.

### Construct validity

**Fig. 1:**
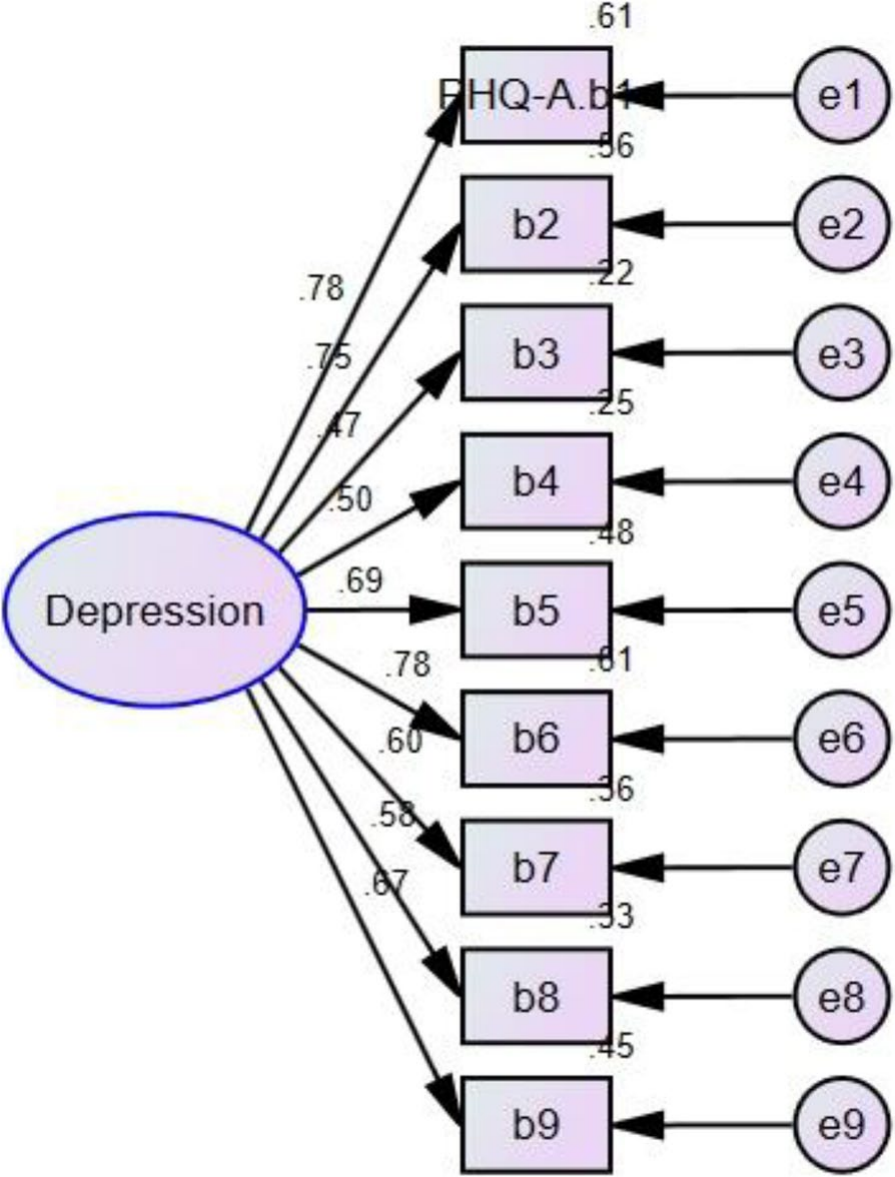
Single-factor CFA analysis to the Chinese translation of the Patient Health Questionnaire for Adolescents (PHQ-A). (Note: b1 = item1 of the PHQ-A; b2 = item2; b3 = item3; b4 = item4; b5 = item5; b6 = item6; b7 = item7; b8 = item8; b9 = item9

AMOS24.0 was used to conduct the confirmatory factor analysis (CFA), which verified the unidimensional structure of the PHQ-A. As demonstrated in Fig. 1, except for the item3 (lamma = 0.47), all items showed relatively adequate loadings on the latent factor (t > 1.96). The goodness of fit indices of the model was within the ideal parameter range. The statistical parameters were as follows: Tucker-Lewis index (TLI) = 0.92, Comparative Fit Index (CFI) = 0.94, Goodness of Fit Index (GFI) = 0.94, Root Mean Square Error of Approximation (RMSEA) = 0.08.

### Reliability analysis

Macdonald Omega coefficient of the PHQ-A was 0.87, demonstrating acceptable internal consistency of the PHQ-A.

The PHQ-A has acceptable test-retest reliability with a Spearman correlation coefficient of 0.70 (*p* < 0.01), showing acceptable stability across time within the scale [43].

### Item-total correlation analysis of the PHQ-A

Pearson correlation analysis was used to calculate the item-total correlation coefficients of the PHQ-A. As demonstrated in Table 2, the item-total correlation coefficients of the scale ranged from 0.45 to 0.71 (*p* < 0.01). The numerical value of the correlation coefficients between the items varied ranged from 0.29 (item 4 and item 5) to 0.68 (item 1 and item 6), showing that item-total score correlations were all significant (*p* < 0.01).

**Table 2:** Item-total correlation and factor loading for PHQ-A

	Item-total correlation	CFA Factor Loading
Item1 Lack of interest	0.71	0.78
Item2 Depressed mood	0.67	0.75
Item3 Sleeping problems	0.45	0.47
Item4 Appetitive problems	0.49	0.50
Item5 Fatigability	0.64	0.69
Item6 Negative feelings about self	0.69	0.78
Item7 Concentration problems	0.56	0.60
Item8 Psychomotor agitation / retardation	0.56	0.58
Item9 Suicidal ideation	0.61	0.67

Note: *CFA* confirmatory factor analysis

### Concurrent validity

The Pearson correlation coefficient of the PHQ-A with the CES-D and MFQ was 0.87 and 0.85 (*p* < 0.01), respectively. The result demonstrated that the PHQ-A has good concurrent validity with these scales.

The patients were classified into different groups by the score of the iCGI-S. Their scores ranged from 1 to 6 (No patient was rated as 7). As shown in Table 3, the Kruskal-Wallis H Test revealed that on the total scores of the PHQ-A, Group 1 was significantly smaller than Group 4, Group 5 and Group 6; Group 3 was significantly smaller than Group 4, Group 5 and Group 6; Group 4 was significantly smaller than Group 5 and Group 6. The results demonstrated that the PHQ-A was able to successfully discriminate patients with different severity of depressive symptoms.

**Table 3:** Kruskal–Wallis H Test among subgroups with different iCGI-S scores

Group comparison	Std.TS	SD	*p* value
group1-group2	-0.519	39.396	1.000
group1-group3	-1.087	22.201	1.000
group1-group4	-4.713	18.497	**0.000**
group1-group5	-8.898	17.653	**0.000**
group1-group6	-6.316	30.182	**0.000**
group2-group3	-0.095	38.785	1.000
group2-group4	-1.813	36.387	1.000
group2-group5	-3.755	36.384	**0.003**
group2-group6	-3.881	43.854	**0.002**
group3-group4	-3.670	17.182	**0.004**
group3-group5	-8.172	16.269	**0.000**
group3-group6	-5.665	29.394	**0.000**
group4-group5	-6.549	10.674	**0.000**
group4-group6	-3.874	26.707	**0.002**
group5-group6	-1.285	26.129	1.000

Note: *Std.TS* Standard test statistic, *SD* Standard Deviation

Values in boldface indicate statistical significance

### Cut-off value

**Fig. 2:**
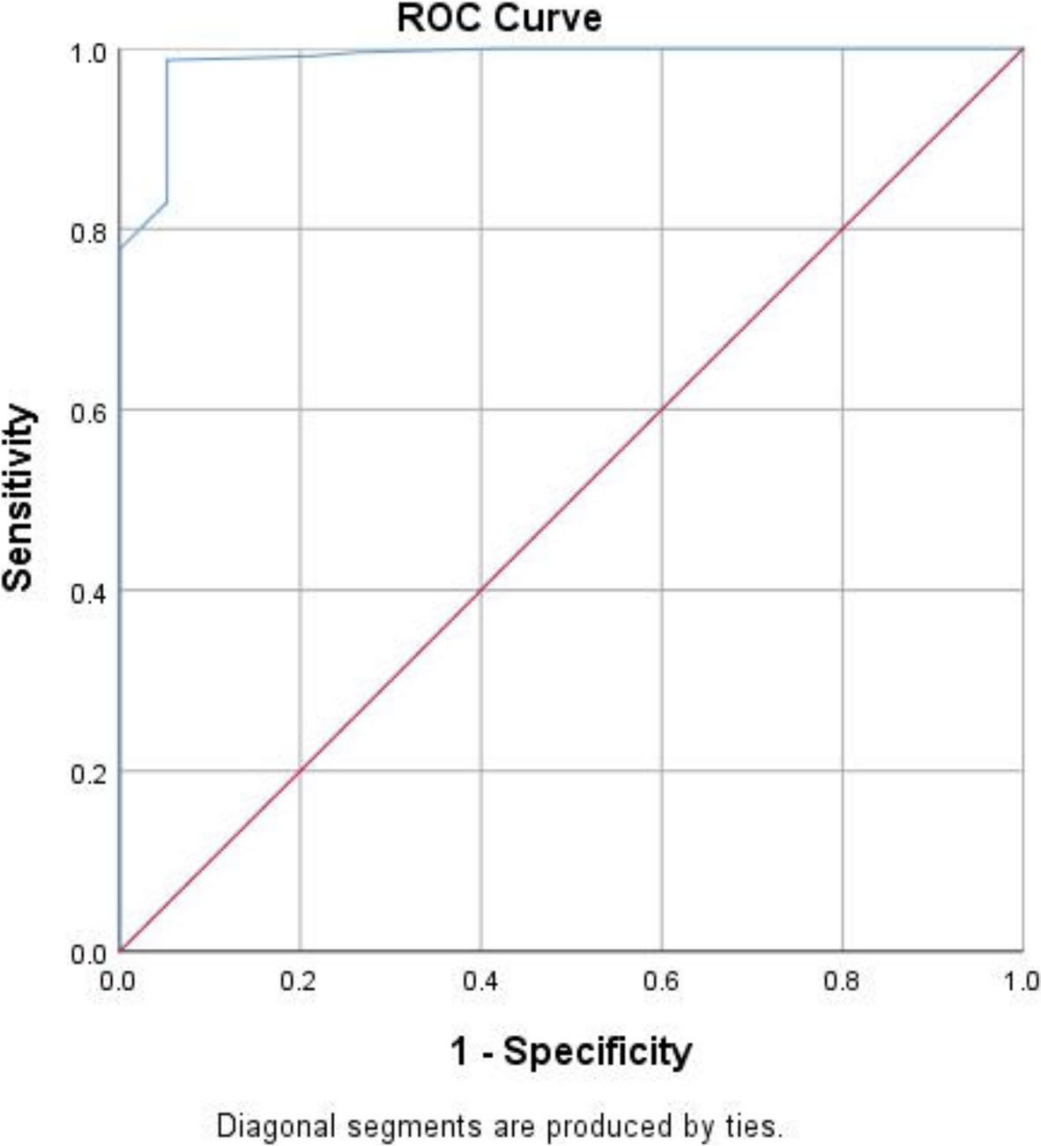
The receiver operating characteristic (ROC) curve of the Chinese translation of the Patient Health Questionnaire for Adolescents (PHQ-A)

Remission of symptoms is the ideal result of treatment for mood disorders, signifying patient recovery from mood disorders [44, 45]. We took iCGI-S = 1 as the criteria for MDD remission and SPSS 25.0 was used to draw the ROC curve of the PHQ-A. As shown in Fig. 2, the areas under the ROC curve (AUC) of the scale were 0.99 (95% CI: 0.97–1.00, *P* < 0.01). The maximum Youden’s index was 0.93, identifying the best cutoff value of 7.5. While the cutoff value was used as an integer, it should be recommended to be 7 or 8 for the PHQ-A. 7 could be a stricter criterion for remission which was clinically helpful. At this point with a sensitivity of 98.7% and specificity of 94.7%, the scale had the best distinction ability.

## Discussion

We investigated the psychometric properties of the PHQ-A among Chinese children and adolescents with MDD in the study.

The CFA showed that except for the item 3 (Sleeping problems), all items showed relatively adequate loadings on the latent factor. This finding of the relatively lower loading on item 3 was consistent with the study of the Jordan version of the PHQ-A [26], suggesting that the item 3 might be less predictive. Generally, all the goodness-of-fit indices of the model fell within the acceptable parameter range, indicating acceptable structural validity of the scale. The CFA of the PHQ-A confirmed its unidimensional structure, which is consistent with previous research findings [26–29].

In terms of the reliability of the PHQ-A, the internal reliability was examined by Macdonald Omega coefficient. The value of the coefficient was 0.87, indicating good internal consistency and high item homogeneity within the PHQ-A. These findings are consistent with previous research on the Arabic, Urdu, Mozambique, and Thai versions of the PHQ-A [26–29]. The Spearman correlation coefficient of the scale was 0.70, which suggested good test–retest reliability for the scale, indicating the stability of the PHQ-A. These findings align with prior studies [27–29]. The satisfactory internal reliability and fair test-retest reliability of the PHQ-A confirmed its acceptable reliability among Chinese adolescents with MDD.

For criterion validity, the CES-D and MFQ were selected as the criterion scales. The total score of the PHQ-A was highly positively correlated with CES-D and MFQ, indicating that the PHQ-A had good criterion validity with them. The results of the Kruskal-Wallis H Test demonstrated that the PHQ-A could successfully discriminate different levels of severity of depressive symptoms among Chinese children and adolescents, consistent with the results of earlier research [25, 26, 28, 29].

This study showed that the area under the ROC curve (AUC) was 0.99, suggesting that the PHQ-A might be a valid tool. However, the AUC of the Thai and Mozambique versions were 0.88 and 0.85 respectively [27, 29]. The very high AUC value in this study might be due to the small sample size of remission (*N* = 19). The optimal cut-off score for the PHQ-A was 7 (7 will be a stricter criterion for remission which is clinically helpful), conforming with the recommended scores from previous studies on other PHQ-A versions [25, 26, 28, 29]. Both the Thai and Mozambique versions of the PHQ-A had an optimal cutoff value of 8. The sensitivity and specificity of the Thai version of PHQ-A were 76% and 81% [29]. The sensitivity and specificity of the Portuguese version were 78% and 80% [27]. Both were lower than the results in our study.

The results indicated that the psychometric properties, the factor structure and the optimal cut-off score of the PHQ-A performed relatively stable across different cultures and languages. There are several limitations in this study. Firstly, since our study focused on only MDD patients, its findings might not apply to other clinical population. Secondly, the sample was recruited from a single mental health center, which was less ideal than multi-center recruitment. Thirdly, this research is cross-sectional, which is unable to confirm whether the scale is sensitive to changes of symptoms after treatment. Finally, the relatively small sample size of remission might cause bias in AUC. Therefore, future studies are needed to test the psychometric characteristics of the PHQ-A in different populations and verify its sensitivity to treatment changes.

## Conclusion

The PHQ-A has satisfactory psychometric properties on Chinese children and adolescents with MDD. It is a simple, reliable, and valid measuring tool for screening and assessing the severity of MDD symptoms among children and adolescents. In this study, it is recommended that the optimum cut-off value of the scale is 7.

## Data Availability

The datasets generated and analyzed during the current study are not publicly available due to limitations written into the participants’ consent forms. Group level data are available from the corresponding author on reasonable request.
